# Gender differences in risk factors for suicide attempts among young, first-episode and drug-naive major depressive disorder patients with anxiety symptoms

**DOI:** 10.3389/fpsyt.2024.1424103

**Published:** 2024-08-08

**Authors:** Donghong Jiang, XiaoE Lang, Dongmei Wang, Xiang-Yang Zhang

**Affiliations:** ^1^ School of Psychology, Shenzhen University, Shenzhen, China; ^2^ Psychological Counselling Centre, Shenzhen University, Shenzhen, China; ^3^ Department of Psychiatry, First Hospital of Shanxi Medical University, Taiyuan, China; ^4^ Key Laboratory of Mental Health, Institute of Psychology, Chinese Academy of Sciences (CAS), Beijing, China; ^5^ Department of Psychology, University of Chinese Academy of Sciences, Beijing, China; ^6^ Affiliated Mental Health Center of Anhui Medical University, Hefei, China; ^7^ Hefei Fourth People’s Hospital, Hefei, China; ^8^ Anhui Mental Health Center, Hefei, China

**Keywords:** gender differences, suicide attempts, major depressive disorder, comorbid anxiety with major depressive disorder, young

## Abstract

**Background:**

Suicide attempts and anxiety are common commodities in patients with major depressive disorder (MDD), and suicide attempts are often associated with anxiety symptoms. Studies have found gender differences in several aspects of MDD; however, gender differences in suicide attempts in young first-episode and drug-naive (FEDN) MDD patients with anxiety remain unknown. This study aimed to investigate potential gender differences in the prevalence of suicide attempts and associated risk factors among young FEDN MDD patients with anxiety in a Chinese Han population.

**Methods:**

A cross-sectional study was conducted on 1289 young patients with FEDN MDD. Demographics, clinical characteristics, and biochemical parameters of patients were collected.

**Results:**

Suicide attempters accounted for 23.80% and 26.12% of male and female FEDN MDD patients with anxiety, respectively, with no significant gender differences. Binary logistic regression analyses showed that anxiety, clinical global impression severity, and thyroid peroxidase antibody significantly predicted suicide attempts in both male and female FEDN MDD patients with anxiety, while body mass index significantly predicted suicide attempts only in males, and psychotic symptoms predicted suicide attempts only in females.

**Conclusion:**

The present study represents the first large-scale investigation of gender differences in the prevalence of suicide attempts and related risk factors among young FEND MDD patients with anxiety in the Chinese Han population. The results indicate that risk factors associated with suicide attempts vary by gender among young FEND MDD patients with anxiety, although a comparable rate of suicide attempts was observed in both female and male patients.

## 1 Introduction

Major depressive disorder (MDD) is a serious public health problem (1) with a high incidence of lifetime suicide attempts (2), which may be higher in younger MDD populations (3–5). Furthermore, female MDD patients have higher rates of suicide attempts than male patients (6, 7), and this gender difference may be more pronounced in younger MDD patients (8). In addition, it has been shown that individuals with MDD who have comorbid anxiety symptoms are more likely to attempt suicide (9). While general gender differences in suicide attempts among individuals with MDD have been studied extensively, gender differences in suicide attempts among young MDD patients with anxiety symptoms have received less attention. The aim of this study was to examine potential gender differences in the prevalence and associated risk factors for suicide attempts in young MDD patients with anxiety. There is an urgent need for this study, which could help to develop gender-specific suicide interventions and prevention strategies for young MDD patients with anxiety symptoms.

Patients with MDD frequently present with anxiety symptoms. In patients with first-episode and drug-naive (FEDN) MDD, the prevalence of anxiety symptoms was found to be 79.2% (10). There are conflicting results regarding gender differences in MDD with anxiety. In some studies, no gender differences were found in the prevalence of anxiety symptoms in MDD patients. For example, a study of 174 Chinese patients with MDD reported no gender difference in the prevalence of comorbid anxiety disorders: 81.20% for women and 79.07% for men (11). A recent study of a large sample of FEDN MDD patients found that the prevalence of comorbid anxiety symptoms in male MDD patients was comparable to that in female MDD patients (80.8% vs. 80.1%) (12). However, other studies have found significant gender differences in the prevalence of anxiety symptoms in MDD patients. For example, a significantly higher proportion of women than men with MDD are reported to have comorbid anxiety (13, 14). A study of Chinese MDD patients also found that female MDD patients were more likely to have comorbid anxiety (58.14% in men vs. 82.78% in women) (15). Furthermore, among young MDD patients, women are more likely to have comorbid anxiety symptoms than men. For example, one study found that young female college students had significantly higher anxiety scores than male college students in their first and second years, while there were no significant gender differences in mean depression scores (16). However, research on gender differences in young MDD patients with anxiety symptoms remains relatively sparse.

Studies have confirmed that anxiety symptoms increase the risk of suicide attempts in MDD patients (9). Furthermore, anxiety may also play an important role in predicting suicide attempts in young adults (17). Regarding gender differences in suicide attempts among MDD patients with anxiety symptoms, there are inconsistent results. For example, in a cohort study involving 50,692 Norwegians, men were found to be more than twice as likely to commit suicide as women when they had comorbid anxiety and depression (18). In contrast, a recent study of FEDN MDD outpatients in China found that among MDD patients with anxiety, more women than men attempted suicide (67.5% vs. 32%) (9). Overall, although studies have shown that anxiety increases suicide attempts in MDD patients, there is a lack of research on gender differences in suicide attempts in young MDD with anxiety symptoms.

Suicide attempts in MDD patients have also been associated with metabolic dysfunction (19, 20) and thyroid hormones (21–23). Low-density lipoprotein (LDL) cholesterol and total cholesterol (TC) levels (20), triglyceride (TG), glucose levels (19), and free triiodothyronine (FT3) levels have been found to differ between suicide attempters and non-suicide attempters among MDD patients (24). It has been demonstrated that metabolic dysfunction in individuals with MDD may be associated with disturbances disruption to biological rhythms (sleep, social, activities, and eating pattern) (25), which may lead to increased depression and thus increase the risk of suicide. Our previous study found that serum thyroid-stimulating hormone (TSH), antithyroglobulin (TgAb), and thyroid peroxidase antibody (TPOAb) levels were higher in MDD patients who attempted suicide than in MDD patients who did not attempt suicide (23). MDD patients with comorbid anxiety had more psychotic symptoms, higher serum levels of TSH, TPOAb, and TgAb, and more suicide attempts (26). Furthermore, studies have shown that there are gender differences in glucose metabolism (27, 28), and also in the relationship between glucose and suicide attempts (29). Thyroid hormones have been shown to play an important compensatory role in preventing further exacerbation of depressive symptoms. However, the onset of hypothyroidism, as indicated by elevated levels of thyrotropin (TSH) and TPOAb (30, 31), may render this compensatory mechanism inadequate and further exacerbate depressive symptoms, thereby increasing the risk of suicide. In addition, thyroid hormone levels in MDD patients appear to vary by gender. For example, female MDD patients have been found to be more likely to have comorbid thyroid dysfunction (32). However, whether there are gender differences in the relationship between suicide attempts and abnormalities in metabolic function or thyroid hormones in young MDD patients with anxiety symptoms are still unclear.

Although studies have shown that anxiety symptoms increase suicide attempts in MDD (9, 17), it is unclear whether this increased risk differs between young men and young women with MDD, what other factors may be associated with suicide attempts in young MDD patients with anxiety, and whether these factors differ between young men and young women. To our knowledge, there are no studies on gender differences in the prevalence of suicide attempts and associated factors in young MDD patients with anxiety symptoms in the Chinese population. It was hypothesized that there would be gender differences in the prevalence and clinical features of suicide attempts and risk factors for suicide attempts in young Chinese FEDN MDD patients with anxiety symptoms. Therefore, the main objectives of this study were to examine gender differences in (1) the prevalence and clinical features of suicide attempts and (2) risk factors for suicide attempts in young Chinese FEDN MDD patients with anxiety. This study is the first large-scale investigation of gender differences in the prevalence of suicide attempts and associated risk factors among young FEDN MDD patients with anxiety in a Han Chinese population. The findings of this study will contribute to the development of gender-specific prevention strategies for suicide attempts in young Chinese FEDN MDD patients with anxiety symptoms.

## 2 Methods

### 2.1 Participants

In this cross-sectional study, participants were recruited from the psychiatric outpatient clinic of a general hospital in Taiyuan, Shanxi Province, China, from 2015 to 2017. Approval for the study was granted by the Institutional Review Board (IRB) of the First Hospital of Shanxi Medical University (No. 2016-Y27). Each participant in the study signed an informed consent form.

The following criteria were used to identify patients with MDD: Han Chinese, aged 18 to 45 years (33, 34); verified the fifth edition of the Diagnostic and Statistical Manual (DSM-V) diagnosis of MDD; HAMD-17 score of 24 or higher; no previous use of antidepressants or antipsychotics; duration of illness less than 24 months; and no previous use of thyroxine or any other specific medication. Patients who met any of the following requirements were disqualified. (1) other psychiatric disorders on Axis I; (2) substance or alcohol abuse or dependence; (3) complications of other physical illnesses; (4) pregnancy or breastfeeding; and (5) refusal to participate in the study.

### 2.2 Clinical interview and assessment

Patient demographic data such as age, gender, education level, marital status, duration of illness, age of onset, and body mass index (BMI) were collected for the study, in addition to clinical data such as blood pressure and suicide attempts. In addition, the researchers reviewed complete medical records and interviewed with family members to clarify unclear information and missing data.

In this study, suicide attempts were defined as self-destructive behaviors in which an individual attempts to end his or her life but does not die (35). Suicide assessment was conducted by two experienced psychiatrists who interviewed each patient. First, all participants were asked, “Have you ever attempted suicide in your life?” All participants who answered “yes” were categorized as suicide attempters, otherwise as non-suicide attempters. In addition, patients were asked about suicide attempts in the past two weeks, the past six months, and the past year. They were then asked for specific information about the suicide attempt, such as the number, timing, and method of suicide, and if the answer was unclear, additional interviews were conducted with their family or friends to confirm. The specific information about suicide attempts was recorded during the interview.

To assess depression, anxiety, and psychotic symptoms and severity of illness, the Hamilton Depression Scale 17 (HAMD-17), Hamilton Anxiety Scale (HAMA), positive subscales of the Clinical Positive and Negative Syndrome Scale (PANSS), and the Global Impression Severity Inventory (CGI) were used. Patients with an initial HAMD score of 24 or more were considered to have major depression (36), while those with a HAMA score of 18 or more were considered to have severe anxiety symptoms (37). Psychotic symptoms were defined as a total score of more than 15 on the PANSS positive subscale (21, 38). Quantitative scores were provided by two psychiatrists with at least 5 years of clinical experience based on HAMD-17, HAMA, and PANSS clinical examinations. The interobserver correlation coefficients between the two independent raters for the three clinical rating scales mentioned above ranged from 0.82 to 0.85.

### 2.3 Measurement of physical and biochemical parameters

On the following morning, blood samples were drawn after an overnight fast and immediately sent to the hospital laboratory for analysis. Total cholesterol (TC), triglycerides (TG), high-density lipoprotein cholesterol (HDL-C), low-density lipoprotein cholesterol (LDL-C), and antithyroglobulin (TgAb), free triiodothyronine (FT3), free thyroxine (FT4), thyroid peroxidase antibody (TPOAb), and thyroid stimulating hormone (TSH) were measured in this study. The Roche C6000 Electrochemiluminescence Immunoassay Analyzer (Roche Diagnostics, Indianapolis, IN, USA) was used to assess the blood levels of FT3, FT4, TSH, TgAb, and TPOAb in the blood. The COBAS C8000 Modular Analyzer (Roche Diagnostics, Mannheim, Germany) was used to measure the levels of TC, HDL-C, LDL-C, and TG using standard laboratory procedures.

### 2.4 Statistical analysis

IBM SPSS 26.0 statistical software was used to analyze the data for this investigation. First, we estimated gender differences in the prevalence of severe anxiety symptoms in young, FEND patients with MDD. Separate chi-squared test (for categorical variables), Mann-Whitney U test (for non-normal variables), and analyses of variance (ANOVA) (for normal and continuous variables) were performed in the male and female groups to compare the differences in clinical parameters between the subclinical groups with and without anxiety. Second, to compare gender differences in the prevalence, demographic, and clinical characteristics of suicide attempts in young FEDN MDD patients with anxiety, 2 × 2 ANOVAs with suicide attempt (2 levels: with suicide attempt and without suicide attempt) and gender (2 levels: male and female) were also performed. Further, separate chi-squared test, the Mann-Whitney U test, and ANOVAs were performed in the male and female groups to compare the differences in demographic, clinical, and laboratory parameters between the subclinical groups with and without suicide attempt. Those significantly different factors were then entered into a logistic regression analysis (backward: Wald) to examine the variables associated with suicide attempts among FEDN MDD patients with anxiety symptoms in both the male and female groups. All p-values were two-tailed with a significance level of ≤ 0.05. Bonferroni correction was used to adjust for multiple comparisons.

## 3 Results

### 3.1 Gender differences in the prevalence of anxiety symptoms among young FEND MDD patients

A total of 1289 young FEND MDD patients were recruited according to the inclusion criteria. Of those young MDD patients, 70.60% (910) were found to have comorbid anxiety symptoms. However, there was no gender difference in the prevalence of comorbid anxiety disorders, 70.64% (332/470) in men and 70.57% (578/819) in women (χ^2^ < 0.001, *p* = 0.98).

This study further explored the gender differences in demographic, clinical and biochemical indicators among young MDD patients with and without anxiety symptoms. **Table 1** shows the demographics, clinical characteristics and biochemical data of males and females with and without anxiety among the young FEND MDD patients. Among male patients, the number of MDD patients with and without anxiety symptoms was 332 (70.64%) and 138 (29.36%), respectively. Compared to male MDD patients without anxiety symptoms, male MDD patients with anxiety symptoms had higher scores on HAMD, HAMA and CGI (*p*’s < 0.001), higher rates of suicide attempts and psychotic symptoms (*p*’s < 0.001), higher systolic and diastolic blood pressure (*p*’s < 0.01), and higher levels of TSH, TC, TPOAb, LDL-C (*p*’s < 0.001), TgAb and TG (*p*’s < 0.05). Of the female patients, 578 (70.57%) had comorbid anxiety symptoms and 241 (29.42%) did not. Patients with anxiety symptoms had higher HAMD, HAMA, and CGI scores (*p*’s < 0.001), higher rates of suicide attempts and psychotic symptoms (*p*’s < 0.001), higher systolic (*p* < 0.001) and diastolic blood pressure (*p* < 0.01), and higher levels of TSH, TgAb, TC, TPOAb, and LDL-C, TG (*p*’s < 0.001) and FBG (*p* < 0.05), but lower HDL-C (*p* < 0.01). After Bonferroni correction, all these differences remained significant (all corrected *p* < 0.05).

**Table 1:** Demographic, clinical, and biochemical characteristics in young FEND MDD patients with and without anxiety, grouped by gender.

	Males(*n* = 470)		*F*/U/χ^2^ (P-value)	Females(*n* = 819)		*F*/U/χ^2^ (*p*-value)
	Anxiety (*n* = 332)	Non-anxiety (*N* = 138)		Anxiety (*n* = 578)	Non-anxiety (*n* = 241)	
Age (years)	28.87 ± 8.80	27.57 ± 8.24	2.200 (0.139)	29.60 ± 8.51	30.10 ± 9.05	0.576 (0.448)
Education duration (years)	13.25 ± 2.73	13.22 ± 2.48	0.015 (0.904)	13.35 ± 2.87	13.41 ± 2.96	0.076 (0.783)
Not married/married	133/199	59/79	0.293 (0.589)^a^	205/373	90/151	0.260 (0.610)^a^
BMI(kg/m2)	24.43 ± 2.14	24.28 ± 2.00	0.475 (0.491)	24.32 ± 1.91	24.34 ± 1.72	0.016 (0.901)
Age at onset (years)	28.72 ± 8.72	27.41 ± 8.10	2.270 (0.133)	29.44 ± 8.41	29.95 ± 8.98	0.598 (0.439)
Duration of illness (months)	5.66 ± 4.09	5.61 ± 4.27	0.017 (0.897)	5.71 ± 4.04	5.52 ± 09.6	0.363 (0.547)
HAMD	31.00 ± 2.79	28.18 ± 2.31	109.899 (0.000)	30.99 ± 2.69	28.25 ± 2.45	186.887 (0.000)
HAMA	22.06 ± 2.60	16.78 ± 1.08	530.725 (0.000)	22.35 ± 2.67	16.80 ± 1.07	974.321 (0.000)
CGI	6.05 ± 0.77	5.54 ± 0.59	47.341 (0.000)	6.10 ± 0.75	5.61 ± 0.61	80.397 (0.000)
Psychotic Symptoms (yes)	33	0	14.753 (0.000)^a^	73	1	30.876 (0.000)^a^
Suicide Attempters (yes)	79	3	31.640 (0.000)^a^	151	11	49.826 (0.000)^a^
TSH, uIU/mL	5.20 ± 2.677	4.23 ± 1.74	16.044 (0.000)	5.26 ± 2.76	4.23 ± 1.81	28.570 (0.000)
TgAb, IU/L	89.36 ± 194.62	52.43 ± 106.56	-2.960 (0.003)^b^	106.82 ± 270.64	42.09 ± 80.17	-5.137 (0.000)^b^
TPOAb, IU/L	85.76 ± 177.17	32.63 ± 73.44	-4.380 (0.000)^b^	79.44 ± 166.19	43.07 ± 142.28	-5.403 (0.000)^b^
FT3, pmol/L	4.95 ± 0.74	5.00 ± 0.74	0.596 (0.441)	4.91 ± 0.72	4.86 ± 0.76	0.946 (0.331)
FT4, pmol/L	16.74 ± 3.02	16.73 ± 2.97	1.066 (0.302)	16.67 ± 3.20	17.00 ± 3.12	0.003 (0.960)
TC, mmol/L	5.38 ± 1.08	4.76 ± 0.87	35.573 (0.000)	5.36 ± 1.12	4.81 ± 1.01	42.847 (0 .000)
HDL-C, mmol/L	1.21 ± 0.30	1.26 ± 0.27	3.324 (0.069)	1.22 ± 0.29	1.28 ± 0.25	7.212 (0.007)
TG, mmol/L	2.20 ± 1.04	2.00 ± 0.90	3.884 (0.049)	2.22 ± 0.99	2.12 ± 0.99	1.548 (0.214)
LDL-C,mmol/L	3.03 ± 0.88	2.69 ± 0.76	15.949 (0.000)	3.06 ± 0.89	2.69 ± 0.75	7.212 (0.007)
Systolic BP, mmHg	117.5 ± 9.91	115.21 ± 9.91	9.906 (0.002)	117.44 ± 10.85	114.40 ± 9.24	7.579 (0.006)
Diastolic BP, mmHg	76.02 ± 6.61	73.96 ± 6.21	9.732 (0.002)	75.73 ± 6.09	74.30 ± 5.85	9.595 (0.002)
FBG	5.38 ± 0.67	5.28 ± 0.55	2.387 (0.123)	5.42 ± 0.64	5.31 ± 0.60	5.864 (0.016)

^a^ represents for using χ^2^ test; ^b^ represents for using U test; HAMD, Hamilton rating Scale for Depression; HAMA, Hamilton anxiety rating Scale; CGI, Global Impression Severity Scale; BMI, Body mass index; TSH, thyroid stimulating hormone; TgAb, anti-thyroglobulin; TPOAb, thyroid peroxidases antibody; FT3, Free triiodothyronine; FT4, Free thyroxine; TC, Total cholesterol; TG, Triglycerides; HDL, High-density lipoprotein cholesterol; LDL, Low-density lipoprotein cholesterol; BP, Blood pressure; FBG, Fasting blood glucose.

### 3.2 Gender differences in the prevalence, demographic and clinical features of suicide attempts in young FEDN MDD patients with anxiety

Among young MDD patients with anxiety symptoms, the suicide attempt rate was 23.80% in male patients and 26.12% in female patients, without significant gender difference (χ^2^ = 0.606, *p* = 0.436 > 0.05). **Table 2** shows the demographic, clinical, and biochemical data on the presence and absence of suicide attempts in males and females with young FEND MDD patients with anxiety.

**Table 2:** Demographic, clinical, and biochemical characteristics of female and male patients with and without suicide attempt among young FEND MDD patients with anxiety.

Characteristic	Males(*N* = 332)		Females(*N* = 578)		Gender F/U/χ^2^ (*p*-value)	suicide attempt F/U/χ^2^ (*p*-value)	Gender ×suicide attempt F/U/χ^2^ (*p*-value)
	Patients with SA (*n* = 79)	Patients without SA (*n* = 253)	Patients with SA (*n =* 151)	Patients without SA (*n* = 427)			
Age (years, M ± SD)	28.28 ± 8.266	29.06 ± 8.97	30.42 ± 7.80	29.30 ± 8.74	3.015(0.083)	0.063(0.802)	1.899(0.169)
Education duration (years, M ± SD)	13.67 ± 2.78	13.12 ± 2.71	13.20 ± 3.24	13.40 ± 2.73	0.174(0.677)	0.597(0.440)	2.817(0.094)
Not married/married	29/50	104/149	56/95	149/278	1.905(0.167)^a^	0.005(0.946)^a^	
BMI(kg/m2)	23.90 ± 2.94	24.59 ± 1.80	24.53 ± 2.18	24.25 ± 1.80	0.747(0.388)	1.632(0.202)	9.347(0.002)
Age at onset (years, M ± SD)	28.08 ± 8.22	28.92 ± 8.88	30.24 ± 7.70	29.16 ± 8.64	3.112(0.078)	0.031(0.861)	1.985(0.159)
Duration of illness (months, M ± SD)	6.28 ± 4.66	5.47 ± 4.06	6.40 ± 4.51	5.46 ± 3.82	0.030(0.862)	7.159(0.008)	0.044(0.833)
HAMD	32.27 ± 3.15	30.60 ± 2.54	32.46 ± 2.43	30.48 ± 2.58	0.024(0.877)	76.607(0.000)	0.585(0.445)
HAMA	23.44 ± 3.11	21.63 ± 2.26	24.03 ± 3.08	21.76 ± 2.23	3.256(0.072)	105.676(0.000)	1.290(0.256)
CGI	6.37 ± 0.75	5.94 ± 0.74	6.58 ± 0.66	5.93 ± 0.71	2.901(0.089)	87.912(0.000)	3.861(0.050)
Psychotic Symptoms (yes/no)	17/62	16/237	34/117	39/388	1.483(0.223)^a^	33.134(0.000)^a^	
TSH, uIU/mL	6.62± 2.58	4.76 ± 2.54	6.75 ± 3.01	4.74 ± 2.47	0.084(0.772)	87.217(0.000)	0.143(0.706)
TgAb, IU/L	110.32 ± 97.28	82.82 ± 193.711	153.76 ± 316.81	90.22 ± 250.62	0.567(0.571)^b^	5.737(0.000)^b^	
TPOAb, IU/L	167.86 ± 82.51	60.121 ± 117.26	155.53 ± 255.89	52.53 ± 107.60	-0.438(0.448)^b^	-5.644(0.000)^b^	
FT3, pmol/L	4.81 ± 0.80	4.99 ± 0.71	4.97 ± 0.78	4.89 ± 0.69	0.228(0.633)	0.727(0.394)	4.904(0.027)
FT4, pmol/L	17.06 ± 3.41	16.54 ± 3.13	16.84 ± 3.28	16.71 ± 2.96	0.016(0.899)	1.726(0.189)	0.614(0.434)
TC, mmol/L	5.80 ± 1.05	5.24 ± 1.06	5.83 ± 1.11	5.19 ± 1.08	0.007(0.932)	48.092(0.000)	0.283(0.595)
HDL-C, mmol/L	1.14 ± 0.30	1.23 ± 0.30	1.13 ± 0.29	1.25 ± 0.28	0.013(0.908)	22.187(0.000)	0.331(0.565)
TG, mmol/L	2.28 ± 1.13	1.02 ± 0.06	2.30 ± 0.92	2.19 ± 1.02	0.044(0.833)	1.763(0.185)	0.005(0.946)
LDL-C,mmol/L	3.19 ± 0.98	2.98 ± 0.84	3.21 ± 0.92	3.00 ± 0.87	0.081(0.776)	8.473(0.004)	0.000(0.993)
Systolic BP, mmHg	120.06 ± 11.57	116.79 ± 9.68	122.37 ± 11.90	115.70 ± 9.90	0.536(0.464)	36.103(0.000)	4.233(0.040)
Diastolic BP, mmHg	77.13 ± 7.98	75.67 ± 6.09	78.19 ± 6.26	74.86 ± 5.79	0.064(0.800)	23.528(0.000)	3.584(0.059)
FBG	5.63 ± 0.69	5.30 ± 0.64	5.55 ± 0.74	5.38 ± 0.61	0.000(0.995)	22.633(0.000)	2.361(0.125)

^a^ represents for using χ^2^ test; ^b^ represents for using U test.

As shown in **Table 2**, two-way ANOVAs with the between-group factors of suicide attempt (2 levels, with suicide attempt and without suicide attempt) and gender (2 levels, male and female) were performed to examine the interaction between suicide attempt and gender. They showed that suicide attempt had a significant effect on HAMD, HAMA, CGI scores, TSH, TC, HDL-C, HDL-C, FBG, and systolic and diastolic BP levels. No significant gender main effect was found (all *p*’s > 0.05). The interaction effects between suicide attempt and gender on BMI (*p* < 0.001), FT3 and systolic BP (*p*’s < 0.05) were significant. Further simple effects analysis showed that the difference on BMI between the subgroups with and without suicide attempts in the female group was not significant, *F* (1, 906) = 2.26, *p=* 0.133 > 0.05, *η*_p_^2^ = 0.002; While the difference on BMI in the male group was significant, *F*(1,906) = 7.23, *p = 0.007* < 0.01, *η*_p_^2^ = 0.08, the BMI of suicide attempters was lower than that of non-attempters in male group. The difference on FT3 between the subgroups with and without suicide attempts in the female group was not significant, *F*(1, 906) = 1.32, *p = 0.25* > 0.05, *η*_p_^2^ = 0.001; while the difference on FT3 in the male group was marginally significant, *F* (1, 906) = 3.62, *p = 0.057*, *η*_p_^2^ = 0.004. The difference on systolic BP between the subgroups with and without suicide attempts in male group was significant, *F* (1, 906) = 6.01, *p* = 0.014 < 0.05, *η*_p_^2^ = 0.007; while the difference in female group was very significant, *F*(1, 906) = 46.403, *p* < 0.001, *η*_p_^2^ = 0.05. Chi-squared test and Mann-Whitney U test were also used to compare the differences in psychiatric symptoms, TPOAb and TgAb between suicide attempters and non-attempters. These differences between the groups were significant (all p’s < 0.001).

### 3.3 Gender differences in risk factors for suicide attempts among young FEND MDD patients with anxiety symptoms

We also analyzed data from the males and females separately to examine risk factors for suicide attempts.

Among male young MDD patients with anxiety group, the number of patients with and without suicide attempts was 79 (23.80%) and 253(77.20%), respectively. Suicide attempters had higher rates of psychiatric symptoms (*p* < 0.001), higher HAMD, HAMA and CGI scores (*p*’s < 0. 001), as well as higher TSH, TPOAb, TC (*p*’s < 0. 001), HDL-C (*p* = 0.013 < 0. 05), FBG (*p* < 0.001), and systolic BP levels (*p* = 0.013 < 0.05), but lower BMI (*p* = 0.012 < 0.05) than non-suicide attempters. After Bonferroni correction, these differences remained significant (all *p*’s < 0.05). Further, a binary logistic regression analysis was performed, with suicide attempts as the dependent variable and the above indicators as independent variables. The results showed that factors associated with suicide attempts included HAMA score (OR = 1.268, 95% CI: 1.121-1.435, *p* < 0.001), CGI score (OR = 1.618, 95% CI: 1.027-2.552, *p* = 0.038 < 0.05), TPOAb (OR = 1.003, 95% CI: 1.001-1.005, p < 0.001), and BMI (OR = 0.799, 95% CI: 0.690-0.925, *p* = 0.003 < 0.01) (**Table 3**).

**Table 3:** Binary logistic regression analyses of determinants of suicide attempts in male MDD patient with anxiety symptoms.

	Coefficients	Std.error	Wald	*p* value	95% confidence interval for EXP (B)		
	B				Exp (B)	Lower	Upper
(constant)	-4.911	3.179	2.386	0.122	0.007		
HAMA	0.237	0.063	14.168	< 0.001^***^	1.268	1.121	1.435
CGI	0.481	0.232	4.297	0.038^*^	1.618	1.027	2.552
TPOAb	0.003	0.001	10.926	0.001^**^	1.003	1.001	1.005
BMI	-0.225	0.075	8.986	0.003^**^	0.799	0.690	0.925

^*^ *p* < 0.05; ^**^ *p* < 0.01; ^***^ *p* < 0.001.

Of the female patients, 151 (26.12%) had and 427 (73.87%) had no suicide attempts. Suicide attempters had higher rates of psychiatric symptoms (*p* < 0.001), a longer duration of illness (*p* < 0.05), higher HAMD, HAMA and higher CGI scores, as well as TSH (*p*’s < 0.001), TgAb, TPOAb, TC, LDL-C, FBG (*p*’s < 0.05), systolic and diastolic BP (*p*’s < 0.001), but lower HDL-C levels (*p <*0.05) than non-suicide attempers. After Bonferroni correction, these differences remained significant (all corrected *p* < 0.05). Further binary logistic regression analysis revealed that the factors associated with suicide attempts were: HAMA score (OR=1.330, 95% CI: 1.217-1.453, *p* < 0.001), CGI score (OR=2.458, 95% CI: 1.685-3.585, *p* < 0.001), TPOAb (OR= 1.003, 95% CI: 1.002-1.004, *p* < 0.001) and psychotic symptoms (OR = 2.250, 95% CI: 1.087-4.654, *p* = 0.029 < 0.05) (**Table 4**).

**Table 4:** Binary logistic regression analyses of determinants of suicide attempts in female MDD patient with anxiety symptoms.

	Coefficients	Std. error	Wald	*p* value	95% confidence interval for EXP (B)		
	B				Exp (B)	Lower	Upper
(constant)	-17.030	2.934	33.699	< 0.001^***^	< 0.001		
HAMA	0.285	0.045	39.647	< 0.001^***^	1.330	1.217	1.453
CGI	0.899	0.193	21.813	< 0.001^***^	2.458	1.685	3.585
TPOAb	0.003	0.001	18.293	< 0.001^***^	1.003	1.002	1.004
Psychoticsymptoms	0.811	0.371	4.779	0.029^*^	2.250	1.087	4.654

^*^ *p* < 0.05; ^***^ *p* < 0.001.

## 4 Discussion

To our knowledge, this is the first large clinical study to examine gender differences in the prevalence of suicide attempts and associated risk factors among young FEDN MDD patients with anxiety symptoms. Key findings of our study included: (1) among young FEND MDD patients, there was no statistically significant difference in the prevalence of anxiety symptoms between males and females (70.64% vs. 70.57%); (2) among young FEND MDD patients with anxiety symptoms, the incidence of suicide attempts was high in both genders, without significant gender differences; and (3) among young FEDN MDD patients with anxiety symptoms, there were gender differences in factors associated with suicide attempts.

In our study, the ratio of female to male patients was 1.74:1, which is similar to previous studies on the gender distribution of other depressed patients (39, 40). However, the present study found no gender difference in the prevalence of anxiety symptoms in young MDD patients (males: 70.64% vs. females: 70.57%), which is similar to our recent study in adult MDD patients (12), but differs from the results of most previous studies. For example, previous studies have found that women report significantly more COVID-19 distress than men (41), and large-scale epidemiological studies have found a higher prevalence of anxiety in female than male MDD patients (14, 42). Furthermore, in a sample of Chinese patients with first-episode depression, women had significantly more comorbid anxiety and sleep problems than men (43). The differences may be due to different sampling frames or cultural backgrounds. For example, in our present study, all participants were young, first-episode, drug-naive patients with MDD with a total HAMD score≥24. Further research is needed on gender differences in MDD patients with anxiety symptoms in other ethnic and clinical groups.

This study reported that the prevalence of suicide attempts among young FEDN MDD patients with anxiety symptoms was over 20% for both genders, which is higher than previous studies (44, 45). To date, only a few cross-sectional studies have reported the incidence of suicide attempts among MDD patients with anxiety symptoms. For example, studies have found the incidence of suicidal ideation (17.9%) in older MDD patients with comorbid anxiety disorders (44). More recently, Xin et al. found that 4.51% of 910 MDD patients with anxiety symptoms or anxiety disorders attempted suicide in the past month (45). The high rate of suicide attempts in MDD patients with anxiety symptoms in our present study may be associated with the untreated MDD patients. Previous studies have suggested that these untreated MDD patients may have a higher risk of suicide (46). In addition, the present study showed that gender differences in suicide attempt rates were not significant among young FEDN MDD patients with anxiety symptoms, whereas previous studies have shown a higher prevalence of anxiety disorders in women (13, 47–49) and a higher rate of suicide attempts in women with MDD (6, 7). This may be because anxiety symptoms are associated with suicide attempts, and gender differences in suicide attempts in MDD patients are offset by gender differences in anxiety symptoms.

The most interesting finding in this study was that there were gender differences in clinical and metabolic factors associated with suicide attempts in young MDD patients with anxiety symptoms. In male MDD patients with severe anxiety symptoms, HAMA score, CGI score, TPOAb, and BMI were significantly associated with suicide attempts, whereas in female MDD patients with severe anxiety symptoms, HAMA score, CGI score, TPOAb, and psychiatric symptoms were significantly associated with suicide attempts. The finding that CGI score was associated with suicide attempts in both male and female MDD patients is consistent with previous finding (35). There are two possible explanations for this finding. One explanation is that suicide attempts are one of the main symptoms in patients with MDD (49), so the more frequent the patient’s suicide attempts, the higher the CGI score, which indicates the severity of the disease (50). Another possibility is that MDD patients with comorbid anxiety symptoms may experience worsening of symptoms that are more difficult to treat (51). Therefore, MDD patients with comorbid anxiety symptoms and higher CGI score may have a higher risk of suicide. This study also found that TPOAb was associated with suicide attempts in both male and female MDD patients, which is consistent with previous studies showing elevated TPOAb in MDD patients with suicide attempts (9) and elevated TPOAb in MDD patients with anxiety (26). This may be because higher TPOAb levels are associated with a higher risk of autoimmune thyroid disease (52), which increases the risk of suicide (53). Furthermore, studies have shown that MDD patients with higher TPOAb levels also have more severe symptoms of depression and anxiety (54, 55), which may increase the likelihood of suicide attempts.

It is known that there are significant gender differences in the psychotic symptoms experienced by people with depression. For example, women with MDD have been reported to be more likely to have comorbid psychosis than men (54, 56). Consistent with these findings, we found that psychotic symptoms were associated with suicide attempts in women with MDD and anxiety symptoms, but not in men. This finding may be associated with hormonal influences. Given the age of young women with MDD, cyclical changes in female sex hormones may exert a considerable impact on mood regulation and the manifestation of anxiety symptoms (57), thereby increasing their risk for suicide attempts.

Surprisingly, our study found that low BMI was associated with suicide attempts only in men with MDD who had anxiety symptoms, but not in women. This is similar to the results of a meta-analysis that found a negative association between obesity and suicide attempts (58), but contradicts previous studies. For example, Branco et al. found an association between obesity and suicide risk in women but not in men (59), and Geulayov et al. found that underweight in middle-aged women was associated with an increased risk of death by suicide (60). This inconsistency may be due to two factors. First, the subjects in this study were diagnosed with MDD with anxiety symptoms, but the population of interest in the Branco et al. study was a large sample of young adults, and the effect of mental disorders on the association between obesity and suicide attempts was not considered in their study. A previous study found that a decrease in BMI was associated with an increased risk of suicide after adjustment for demographic characteristics, mental disorders, and Charlson comorbidity score (61). In addition, a decrease in BMI may lead to a sense of physical loss, which is associated with suicide attempts (40). Second, lower BMI or weight loss is more acceptable in women than in men (62), which may reduce the predictability of suicide attempt risk in women, such that low BMI predicts suicide attempts only in male MDD patients with anxiety. In addition, the finding that BMI was a significant predictor of suicide attempts only in men may be related to societal pressures on male body image. Individuals with MDD tend to have reduced appetite, resulting in a lower BMI. However, young men are expected to have a healthy body image (63). Young men with MDD who have a lower BMI may be more dissatisfied with themselves, which may increase the likelihood of suicide attempts. Our findings contribute to a more nuanced understanding of the role of gender in risk factors for suicide attempts, thereby informing the development of gender-specific intervention strategies.

This study has several limitations. First, due to the cross-sectional nature of the study, these variables can only be considered correlated, not causal. A prospective cohort study is needed to confirm our findings. Second, because our sample was Han Chinese and included only patients with MDD recruited from psychiatric outpatient clinics, our findings need to be validated in other groups with different ethnic and clinical backgrounds. Third, data on suicide were collected through interviews, rather than through the use of a structured scale, such as the Columbia-Suicide Severity Rating Scale (64). This may have influenced the results due to the involvement of subjective factors and should be remedied in future studies.

## 5 Conclusions

In conclusion, our study found no gender differences in the prevalence of suicide attempts among young FEDN MDD patients with anxiety symptoms. Furthermore, lower BMI was associated with suicide attempts only in male MDD patients with anxiety symptoms, whereas having psychotic symptoms was associated with suicide attempts only in female MDD patients with anxiety symptoms. HAMA, CGI scores, and TPOAb were found to be independently associated with suicide attempts in both male and female MDD patients with anxiety symptoms. Understanding these gender differences may facilitate the development of gender-specific diagnostic techniques, as well as treatment and interventions for suicide attempts. However, given the limitations of cross-sectional design in this study, our findings need to be confirmed in further studies using a prospective cohort design and a broader sample.

## Data availability statement

The data analyzed in this study is subject to the following licenses/restrictions: Patient data must be handled with strict confidentiality and privacy measures to protect sensitive information, which could restrict data access and sharing. Requests to access these datasets should be directed to JD, jiangdh@szu.edu.cn.

## Ethics statement

The studies involving humans were approved by the Institutional Review Board (IRB) of the First Hospital of Shanxi Medical University. The studies were conducted in accordance with the local legislation and institutional requirements. The participants provided their written informed consent to participate in this study.

## Author contributions

DJ: Writing – review & editing, Writing – original draft, Validation, Supervision, Methodology, Investigation, Formal analysis, Conceptualization. XL: Writing – original draft, Validation, Resources, Project administration, Data curation, Conceptualization. DW: Writing – original draft, Validation, Resources, Project administration, Investigation, Data curation. XZ: Writing – review & editing, Validation, Supervision, Resources, Project administration, Methodology, Conceptualization.
